# Application of In Vivo Imaging Techniques for Monitoring Natural Killer Cell Migration and Tumor Infiltration

**DOI:** 10.3390/cancers12051318

**Published:** 2020-05-22

**Authors:** Prakash Gangadaran, Ramya Lakshmi Rajendran, Byeong-Cheol Ahn

**Affiliations:** 1 Department of Nuclear Medicine, School of Medicine, Kyungpook National University, Daegu 41944, Korea; prakashg@knu.ac.kr (P.G.); ramyag@knu.ac.kr (R.L.R.); 2 BK21 Plus KNU Biomedical Convergence Program, Department of Biomedical Science, School of Medicine, Kyungpook National University, Daegu 41944, Korea; 3 Department of Nuclear Medicine, School of Medicine, Kyungpook National University Hospital, Daegu 41944, Korea

**Keywords:** natural killer cell, in vivo tracking, migration, infiltration, bioluminescent, MRI, SPECT, PET

## Abstract

In recent years, the use of natural killer (NK) cell-based immunotherapy has shown promise against various cancer types. To some extent therapeutic potential of NK cell-based immunotherapy depends on migration of NK cells towards tumors in animal models or human subjects and subsequent infiltration. Constant improvement in the pharmacological and therapeutic properties of NK cells is driving the performance and use of NK cell-based immunotherapies. In this review, we summarize the molecular imaging techniques used in monitoring the migration and infiltration of NK cells in vivo at preclinical and clinical levels. A review of pros and cons of each molecular imaging modality is done. Finally, we provide our perception of the usefulness of molecular imaging approaches for in vivo monitoring of NK cells in preclinical and clinical scenarios.

## 1. Introduction

Natural killer (NK) cells, a type of lymphocyte, were first reported almost four decades ago. They are granular in nature and are involved in immune-surveillance [1]. NK cells are integral to the innate immune system and therefore, act as the first-line of defense against invading pathogens [2]. NK cells are a promising cell type for adoptive cell therapy for several reasons. They do not require priming or prior sensitization to interact with and kill tumor cells [3,4]. NK cells have a proven ability to detect neoplastic cells in the body, as demonstrated by accumulating evidence over the last four decades. The resulting knowledge about NK cell regulation has improved the effectiveness and safety of the NK cell treatment. Migration of NK cells into tumors is a critical factor for elimination of the aberrant cells [5]. Limited efficiency of NK cells depends on the number of malignant cells that are killed by one NK cell and migration speed of NK cells. These limitations hamper the efficacy of the immune system to the tumor. The tumor microenvironment poses a major challenge to clinical applications of the NK cell due to immune suppressive signals that disturb both tumoral infiltration of NK cells and their activation at the tumor site [6]. However, recent studies have reported the heterogeneity of NK cell populations and variability in host responses to NK cell therapy [7]. Isolation and ex vivo amplification of the most therapeutically efficient NK cell subpopulation from the total population of NK cells from a patient with a tumor could allow better treatment. 

Oncologists have questioned the tumor homing capability of ex vivo expanded NK cells, and their cytotoxicity in the tumor microenvironment. Over the past 20 years, only a few clonal NK-cell lines had been established (NK-92, NK-YS, KHYG-1, NKL, NKG, SNK-6 and IMC-1). The NK-92 line was derived from peripheral blood of 50-year old Caucasian man with non-Hodgkin’s lymphoma [8]. NK-92 cells are cytotoxic to various cancer cell types in vitro and in vivo. These cells are the only ones to date that have a clinical benefit and minimal side effects following their infusion into patients with advanced cancers [9,10]. Efficient immunosurveillance of NK cells requires cell motility and constant surveillance of tumor cells [11,12]. NK cells have an extremely heterogeneous range of migratory behaviors at different stages of development [13]. Fibronectin facilitates the migration of NK cells to tumors and chemokines are important in the recruitment of NK cells [14,15]. Several strategies have significantly increased NK cell migration and infiltration within tumor tissues [16,17]. 

Realizing the therapeutic potential of NK cells requires more knowledge of cell characteristics. Non-invasive in vivo real-time imaging of NK cells in animal tumor models and human subjects become important in this context to determine the NK treatment success and efficacy. In this review paper, we discuss molecular imaging techniques used to evaluate the migration and infiltration of NK cells. We also discuss the advantages and shortcomings of approach. Finally, we review the development of the technologies and designs that has led to the currently available sophisticated methods with specific in vivo examples.

## 2. Non-Invasive In Vivo Imaging Modalities

Precise in vivo imaging of NK cell migration is extremely useful for basic and advanced biomedical researches, and for practical applications. Optical imaging methods are especially well-suited for visualizing infused cells in preclinical animal studies as they are very sensitive and non-invasive [18,19]. Optical imaging that uses visible light can provide multiplex imaging results by analyzing special properties of photons. Both tumor and NK cells can be visualized simultaneously in an animal model. These images have been used by scientists for research and by physicians for diagnosis and cell-based treatments [20,21]. Optical imaging is a powerful method for in vivo real time cell tracking in small animals over time without animal sacrifice [18,22]. Even-though optical imaging possesses many advantages but has the disadvantage of limited tissue penetration depth and it is not suitable for clinics [23]. Optical imaging can be of two main types—fluorescent and bioluminescent imaging. Fluorescent imaging needs external light source to activate protein molecules. Fluorescent dye used for cell labeling may show non-cell-associated signals in tissues or organs even after death of the labeled cells [24]. On the other hand, bioluminescent imaging captures a natural light produced via the interaction between bioluminescent proteins and their substrates such as firefly luciferase and D-luciferin. Bioluminescent imaging requires genetically modified luciferase and this imaging is also not suitable for clinics [25].

Advantages of nuclear imaging using radionuclides are excellent sensitivity and no depth limitation [26]. Compared to optical imaging, nuclear imaging is adept at visualizing deeply located organs [18,27,28]. Therefore, nuclear imaging could be a good candidate for in vivo tracking NK cells and evaluating their therapeutic efficacy in large animals and even in humans. Nuclear imaging also has its own disadvantages such as ionizing radiation exposure, long scan time [20,29]. Some nuclear imaging techniques such as single-photon emission computed tomography (SPECT) or positron emission tomography (PET) can be used to obtain three- dimensional tomographic images data. Magnetic resonance imaging (MRI) has high resolution with no ionizing radiation unlike nuclear imaging. MRI also has disadvantages such as a lower sensitivity than PET or SPECT and an expensive cost [30,31].

Two is better than one, so dual or hybrid imaging using nuclear imaging combined with anatomical imaging, such as computed tomography (CT) or MRI, are clinically available. Therefore, allowing us to examine the functional and also structural imaging at the same time. The hybrid technology allows accurate localization of the NK cells in subjects; MRI with an advantage of excellent resolution for anatomical structures has been successful in this regard as nuclear imaging has low anatomical resolution (PET/SPECT) [32,33,34]. Advancement in the field of imaging technologies are encouraging and forthcoming hybrid imaging systems will reveal accurate in vivo kinetics of NK cells in the near future.

## 3. In Vivo Monitoring of NK Cell Migration and Infiltration into a Tumor by Molecular Imaging

Development of NK cell-based cytotherapy requires a clear understanding of migration followed by the infiltration of NK cells to tumors and an ability to quantitatively analyze their in vivo fate after administration. Noninvasive in vivo imaging modalities for the administered NK cells might permit the comprehensive understanding of the in vivo therapeutic effects of NK cells for diseases including cancerous diseases by providing accurate data of the in vivo distribution and kinetics of the cells. Recent advancements in in vivo molecular imaging permit the recognition of simultaneous multiple biological processes within living organisms [20]. Translation from in vitro to in vivo settings allows real-time visualization of NK cells in animal models and humans [21,27,28,32,35].

Lim et al. studied the in vivo cytotoxicity effects of NK92MI cells labeled with anti-human CD56 antibody coated QD705 (near infrared [NIR]-emitting fluorescent quantum dot) using melanoma tumor-bearing mice. NK92MI cells were retained in the tumors 24 h following intratumor injection as shown tumor by fluorescence imaging. Localization of NK cells in the tumor is one of requisites for antitumor activity. The observed reduction in tumor size suggested the potential therapeutic effect of the administered NK cells. Viability and therapeutic effect of the NK cells were not influenced by the fluorescence imaging agent to NK cells [36]. However, other studies used techniques of lipofection or electroporation to label genetically modified NK cells with iron-oxide contrast agents for MRI [37,38]. However, delivery of the iron-oxide contrast agents into the NK cells by electroporation damages the cell membrane and decreases cell viability [37]. In addition, imaging of ferumoxides labeled NK cells with MR technology showed relatively low sensitivity [38]. Jang et al. demonstrated the controlled migration of NK cells towards human B cell lymphoma using an external magnet in a mouse model. NK92MI cells were labeled with cy5.5-conjugated Fe_3_O_4_.SiO_2_ core/shell nanoparticles to control the migration of the NK cells in the external magnetic field. Fluorescence imaging revealed migration control of NK cells by the technique. The NK cells loaded with nanoparticles migrated to the tumor and infiltrated into the tumor up to 17-fold greater compared to the control. The labeling procedure did not affect the killing activity of the NK cells [39]. Bioluminescent imaging is a promising method for long term NK cell tracking in small animals because its signal will not be diluted by cell division and signal disappears after death of the cells [21] but inverse in NIR dye labeling [39].

Wennerberg et al. demonstrated NK cell migration using a NIR lipophilic dye (DiR) in a xenograft mouse model. The study was based on the knowledge that NK cells express chemokine receptor chemokine receptor 3 (CXCR3), the receptor for CXC ligand 10 (CXCL10). To exploit this chemoattraction, the authors transfected a human melanoma cell line with CXCL10 then implanted the cells into mice. Fluorescence imaging revealed that intravenously injected DiR-labeled NK cells migrated and infiltrated to melanoma expressing CXCL10 rather than to negative melanoma tumor [17]. Tavri et al. described the migration of genetically modified NK cells (NK-92-scFv(MOC31)-zeta cells; scFv: humanized single-chain Fv antibody fragment) by labeling the NK cells with another NIR lipophilic dye (DiD). Fluorescence imaging revealed the migration of the NK cells to tumor-associated epithelial cell adhesion molecule (EpCAM) expressing human prostate cancer cells in a mouse model 90 min-post intravenous injection of the NK cells. Delayed fluorescence imaging revealed that more NK cells migrated to the tumor at 8 and 24 h [40]. Overall, the direct labeling has become a strategy of choice among researchers due to its simplicity and feasibility, even though it has some flaws [18]. Uong et al. demonstrated ex vivo-expanded NK cell migration using ESNF13 NIR fluorophores in the metastatic and xenograft mouse model. Fluorescence imaging successfully visualized intravenously injected ESNF13-labeled NK cells migrating to the tumors. Fluorescence imaging revealed migration of the NK cells to lung metastatic and xenograft breast cancer within 30 min, with the strongest signal was observed at 30 min (lung metastasis) and at 1 h (xenograft) [41]. Lee et al. developed an antibody-based NK-cell–homing protein, named NK-cell–recruiting protein-conjugated antibody (NRP-body). They demonstrated the infiltration of NK cells to tumors in an orthotopic and metastatic pancreatic cancer mouse model. Two weeks post-injection with cancer cells, NRP-body and control-body were administered. NK cells were then intravenously injected into the mice. Fluorescence imaging at 5 days post-injection of NK cells showed that mice injected with NRP-body showed significantly higher NK cells infiltration to orthotopic and metastatic pancreatic cancer than control-body injected mice and reduced the tumor burden and size [42].

Zhu et al. transduced the firefly luciferase (Fluc) gene into NK cells (NK/Fluc) and used bioluminescent imaging to track the migration of intravenously injected cells towards the anaplastic thyroid cancer (CAL-62) in mouse models with lung metastasis or xenograft. Imaging revealed that NK/Fluc cells migrate and infiltrate into the lung tumor within 1 h, remain there for up to 24 h and then reduced in number at 48 h. Furthermore, in a xenograft model, intravenously injected NK/Fluc cells migrated to the lung within 1 h and then migrated to the xenograft tumor within 3 h. The cells remained in the tumor for up to 24 h, with reduced cell number detected at 48 h [21]. Optical imaging features low spatial resolution and poor tissue penetration, which limits its possibility of clinical translation [17,21,36,39].

Other researchers investigated the migration of NK-92-scFv(MOC31)-zeta cells to EpCAM-positive prostate cancers in a rat model using MRI. Ferumoxide was used for labeling to permit their tracking. These cells and ferumoxide-labeled EpCAM-targeting NK-92-scFv(MOC31)-zeta cells were intraperitoneally injected in a prostate tumor-bearing rat. Imaging demonstrated marked negative tumor enhancement 1- and 24-h post-injection of the labeled NK cells. The signal remained unchanged at 1- or 24-h post-injection of the labeled NK-92 cells. The observations clearly indicated that NK-92-scFv(MOC31)-zeta cells migrated to the tumor site killed tumor cells [38]. One flaw in the experimental design was that the long-term imaging was not performed. Another study using MRI demonstrated migration of genetically modified NK-92 (NK-92-scFv(FRP5)-zeta) cells into HER2/neu positive mammary tumors. scFv(FRP5)-zeta is a chimeric antigen receptor specific to the tumor-associated ErbB2 (HER2/neu) antigen. NK-92-scFv(MOC31)-zeta and parental NK-92 cells labeled with ferumoxide and ferucarbotran were intravenously injected into mice harboring mammary tumors in the mammary fat pad. Imaging revealed migration of NK-92-scFv(MOC31)-zeta cells to the tumor. While labeling did not affect the migratory properties of NK cells, electroporation caused impairment of cell viability [37]. Mallett et al. also used MRI to visualize the migration of KHYG-1 human NK cells to prostate tumors in nude mice. Ultra-superparamagnetic iron oxide labeled KHYG-1 cells were subcutaneously injected into the tumor region, this labeling method did not affect the cell viability of KHYG-1 cells. A limitation of the study was that macrophages might take up the iron, which was released from the labeled NK cells [34]. 

A recent study investigated the migration of NK cells by labeling them with ^19^F perfluorocarbon and assessing their tumor infiltration in neuroblastoma mouse model. MRI revealed cell infiltration into tumors at day 0 and 2. The labeled cells were also injected via the same route into mice harboring human mantle cell lymphoma. MRI revealed tumor infiltration of the cells at days 0, 1, 3 and 8. Upon subcutaneous injection of labeled cells into melanoma-bearing mice, tumor infiltration was observed on days 0, 1, 3, 7, 10 and 15 [33]. The labeling procedure did not affect the expression of NK cell cytotoxicity mediators (IFNɣ and granzyme B) and cytotoxicity as well. ^19^F does neither decay nor require phagocytosis to enter cells. It is a component of the FDA approved drugs and long safety records in humans [33,43]. One obvious limitation was non-detectability of ^19^F-labeled NK cells in the lungs due to signal loss via multiple air-tissue interfaces [33]. A new study investigated the infiltration of murine NK cells (LNK) to tumors using MRI by labeling LNK cells with ferumoxytol and tumor infiltration of the NK cells was assessed in mice. Ferumoxytol-LNK cells were injected through hepatic arterial or venous routes in a rat bearing hepatocellular carcinoma. MRI results revealed that LNK cells injected via both routes migrated to the tumor. The hepatic arterial infusion increased the NK cell migration efficacy and decreased the tumor volume compared to intravenous injection [44]. Another study demonstrated the infiltration of SPIO labeled NK-92MI cells into tumors using MRI. The NK cells were infused through the hepatic artery. MRI revealed a higher signal in the tumor than in normal liver tissue, which clearly showed that the NK cells infiltrated to the tumor [32]. MRI has limitations of low sensitivity and slow acquisition time. In addition, ferumoxide labeling can cause cellular damage, which can induce more NK cell death in vivo [32,34,37,38]. Furthermore, MR contrast agents released from NK cells in vivo may lead to non-specific signals from the free contrast agents [32,34].

Other imaging modalities have been used in some studies. Melder et al. used PET imaging to visualize activated NK cell infiltration into fibrosarcoma in a mouse model. Cells were labeled with [^11^C] methyl iodine and some of them were activated by interleukin (IL)-2. Activated and non-activated NK cells were injected into the lateral tail vein distal to the fibrosarcoma. PET imaging revealed no change in the migration of activated and non-activated NK cells at 30 min, while at 60 min a stronger tumor signal was obtained with the activated cells. PET imaging visualized accumulation of activated NK cells localized in small tumors [45]. PET provides advantages of excellent sensitivity and absolute quantification in a non-invasive setting, which is potentially applicable to clinical studies of cell delivery and localization [45,46,47].

SPECT imaging was used to visualize the migration of the allogenic NK cell to a tumor in renal cell carcinoma patients. To get an image of NK cells in the patients, the cells were labeled with ^111^In. Signals were obtained at two out of four large metastases [28]. SPECT imaging was also used to assess the in vivo migration of autologous NK cells to hepatic metastasis in colon cancer patients. Ex vivo expanded NK cells were labeled with ^111^In oxine and injected into hepatic artery of the patients. The injected NK cells were confined to the liver and spleen and accumulation of NK cells in malignant lesions was observed by SPECT. On the contrary, intravenously injected NK cells migrated to the lung, and then to the spleen, not to metastatic tissues [27]. A limitation of the study was the relatively low labeling efficiency of NK cells with ^111^In (less than 60 %) [27].

**Figure 1:**
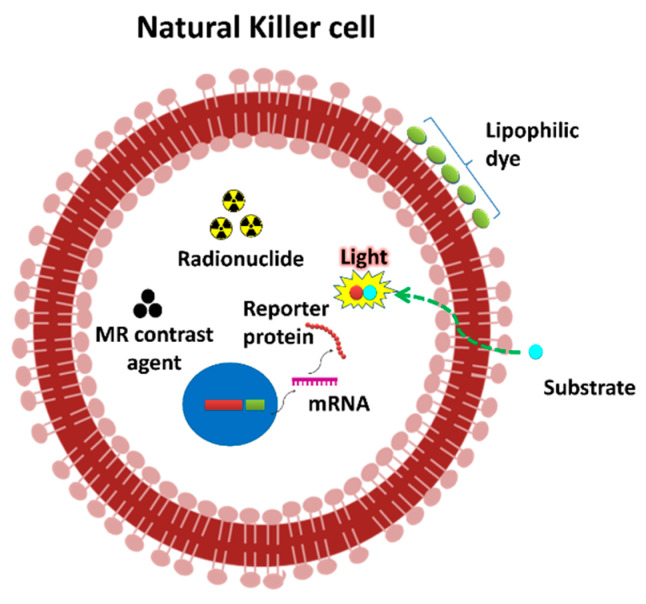
Labeling strategies of the NK cell for non-invasive imaging. At first, reporter genes (Firefly luciferase) are transduced to NK cells. Then, NK cells express the reporter protein. Lipophilic labeling agents (e.g., DiD, DiR and cy5.5) could bind to the membrane of the NK cells. ^111^In-oxine and ^99m^Tc-oxine are lipophilic and penetrate the membrane of cells. [^18^F]FDG can be transported into cells via glucose transporters. NK cells can be electroporated or incubated with molecules such as ultra-small super paramagnetic iron oxide (USPIO).

**Figure 2:**
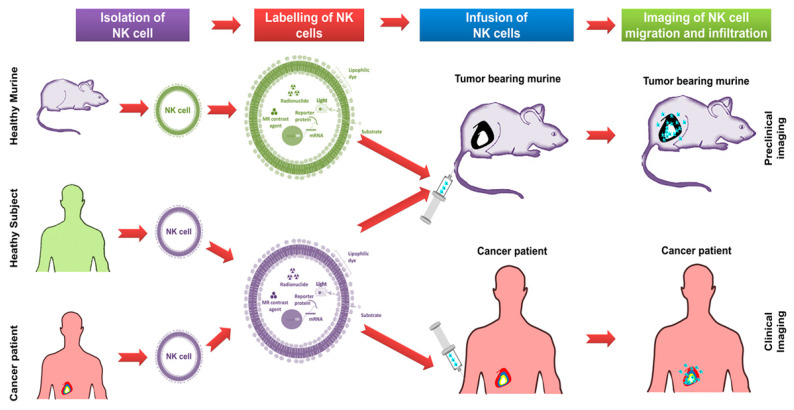
An overview of steps involved in tracking NK cell migration and infiltration using non-invasive in vivo imaging approaches. NK cells obtained from murine or healthy subject or cancer patients; labeling of NK cells with imaging agents; the infusion of NK cells into a tumor-bearing murine model or cancer patient and in vivo imaging of NK cell migration and infiltration by various modalities such as fluorescence and bioluminescence imaging (preclinical) as well as magnetic resonance imaging, positron-emission tomography and computerized tomography (clinical).

In another study, autoradiography was used to assess the migration of [^18^F]fluorodeoxyglucose (FDG)-labeled genetically modified NK-92-scFv(FRP5)-zeta cells to HER2/neu-positive sarcoma in a mouse model. Tumor migration of the genetically modified cells was found to be two-fold higher compared to parental NK cells. Labeling efficiency was 81% after 60 min of incubation and cell viability was unaffected by the labeling protocol. However, in order to carry out NK cell monitoring for a longer duration the strategy should be selected [48]. Finally, Galli et al. demonstrated migration of NK cells using gamma camera imaging of NK cells labeled with ^99m^Tc-anti-CD56 monoclonal antibody. NK cells accumulated in anaplastic thyroid cancer within 24 h post-intravenous injection [35]. Nuclear imaging has slow acquisition time and labeling can be complicated [28,35,48]. Molecular imaging studies showing in vivo migration and infiltration of the NK cell to cancers are summarized in Table 1 and illustrated in Figure 1 and Figure 2.

## 4. Challenges in the NK Cell Expansion and Labeling

In the above reviewed studies, nine of the studies used the primary NK cells, followed by five studies with NK-92MI and four studies with NK-92. In addition, KHYG-1, LNK and murine NK cells were used by one study each (Table 1). Generally, the engineered NK cell lines (NK-92 or NK-92MI) are readily growing cells without Interleukin-2, when compared to primary NK cells [49]. Most preclinical and clinical studies using primary NK cells are derived from peripheral blood mononuclear cells (PBMCs), especially all the studies mentioned in this review. Primary NK cells represent only 10% of the lymphocytes in the blood and obtaining functional NK cells is challenging [50]. Cell lines are mostly suitable for preclinical studies as reported and only NK-92 have been infused to advanced cancers patients with positives results and less side effects [51,52]. Further investigations are needed for successful NK cell-based therapies, such as developing a highly expanding, functional and clinically safe cell lines. Engineering to add chimeric antigen receptor (CAR) to primary NK cells and it revealed improve the therapeutic effects of NK cells and both NK cells and CAR modified NK cells are already in clinics [53,54]. In vivo fluorescent imaging of NK cells was mostly done by labeling of lipophilic dyes, after injection of dye labeled NK cells to animal/humans, division of NK cells dilutes imaging signals, whereas, in vivo optical imaging with transfection of reporter genes (such as luciferases) to NK cells does not reveal the signal dilution by cell division [17,21,42]. Therefore, reporter gene imaging can be suitable for studying the longevity of NK cells in vivo. However, genetic engineering of NK cells with a reporter gene is a laborious and difficult process. On the contrary, fluorescent imaging after direct dye labeling of NK cells is feasible and does not incur any genetics changes, therefore, the technique is suitable for a short-term study of small animals [55,56]. MR contrasts agents labeling also similar to that of fluorescent dye labeling, it could dilute the contrast agent upon cell division and signal persistence after cell death [32,33,34,43], in contrast signals of a reporter gene will disappear after cell death [57]. SPECT and PET imaging by radionuclides labeling to NK cells are available and decay of radioactivity limits long term in vivo imaging of NK cells [56,58].

## 5. Future Prospects of In Vivo NK Cell Imaging

Since each modality has its own limitations, multimodal hybrid imaging consisting of two or more imaging technologies can provide more informative results. Commercial systems integrating optical, PET, SPECT, CT and MR technologies in various combinations are already available. These hybrid techniques allow integration of the strengths of individual modalities. For example, recent advancement in tomographic fluorescence fusion systems allowed us to combine optimal imaging and high-resolution anatomical imaging techniques such as CT or MRI. This way, we can overcome the flaws in optical imaging approaches such as poor resolution. Likewise, a hybrid of PET and MRI can offer high sensitivity of PET and excellent resolution of MRI together, which are clinically relevant characteristics. Therefore, the hybrid imaging can be very useful for in vivo monitoring of NK cells in patients. These emerging developments can accelerate clinical application of promising NK cell-based therapies by accurate in vivo monitoring of administered therapeutic NK cells. Better in vivo NK cell monitoring should be possible with advancement of tracer chemistry and imaging technologies, which will ultimately progress the field of NK cell-based immunotherapy with further improvements in our understanding of genetic regulation in the NK cells.

## 6. Conclusions

Successful NK cell migration and infiltration relies on a variety of factors to ensure that NK cells reach the tumor and are able to eliminate it without being suppressed in the process. Continued studies on the intrinsic mechanisms of NK cell biology and regulation are warranted to develop more safe and efficient therapeutic strategies. Our knowledge of how NK cells migrate to tumors and other components of the immune system will continue to increase with the help of various molecular imaging technologies. This knowledge will contribute to the selection of the most suitable NK cell-based immunotherapy for certain clinical scenarios. Our capacity to design more effective personalized NK cell therapies will likely increase in the near future.

## Figures and Tables

**Table 1:** In vivo monitoring of natural killer (NK) cell migration and infiltration into tumors by molecular imaging techniques.

Imaging	Imaging Modality	Labeling Method/Agent	Cell Type	Naïve/Modified Cell	Subject	Route of Injection	Duration	Migration/Infiltration to Tumor	Clinical Translation	Ref.
Optical Imaging	FLI	NIR dye	NK92MI	Naïve	Mice	Intratumor	24 h	Infiltrated to melanoma	Limited	[36]
		Cy5.5	NK92MI	Naïve	Mice	Intravenous	Immediate	Migrated and Infiltrated to B cell lymphoma	Limited	[39]
		DiR	Primary NK	Naïve	Mice	Intravenous	5 days	Migrated to CXCL10 expressing melanoma	Limited	[17]
		DiD	NK-92	NK-92-scFv(MOC31)-zeta	Mice	Intravenous	1.5, 8 and 25 h	Migrated to EpCAM expressing prostate cancer	Limited	[40]
		DiR	Primary NK	NRP-body	Mice	Intravenous	5 days	Infiltrated to pancreatic cancer	Limited	[42]
		ESNF13	Primary NK	Naïve	Mice	Intravenous	0.5, 1, 2 and 4 h	Migrated to lung metastatic and xenograft breast cancer	Limited	[41]
	BLI	Fluc	NK92MI	Naïve	Mice	Intravenous	1, 3, 24 and 48 h	Migrated to lung metastatic thyroid cancer	Limited	[21]
		Fluc	NK92MI	Naïve	Mice	Intravenous	1, 3, 24 and 48 h	Migrated to xenograft thyroid cancer	Limited	[21]
Magnetic Resonance Imaging	MRI	ferumoxides	NK-92	NK-92-scFv (MOC31)-zeta	Rat	Intraperitoneal	1 and 24 h	Migrated to EpCAM expressing prostate cancer	Yes	[38]
		ferucarbotran	NK-92	NK-92-scFv (FRP5)-zeta	Mice	Intravenous	12 and 24 h	Migrated to HER2/neu positive NIH3T3 mammary tumors	Yes	[37]
		USPIO	KHYG-1	Naïve	Mice	Subcutaneous		Migrated to prostate cancer	Yes	[34]
		^19^F	Primary NK	Naïve	Mice	Intratumor	0–2 days	Infiltrated to neuroblastoma	Yes	[33]
		^19^F	Primary NK	Naïve	Mice	Intratumor	0–8 days	Infiltrated to Mantle cell lymphoma	Yes	[33]
		^19^F	Primary NK	Naïve	Mice	Subcutaneous	0–15 days	Infiltrated to melanoma	Yes	[33]
		SPIO	NK92MI	Naïve	Rat	Intraarterial	Immediate	Infiltrated to hepatocellular carcinoma	Yes	[32]
		ferumoxytol	LNK	Naive	Rat	Intravenous and transcatheter	1, 2 and 8 days	Migrated to hepatocellular carcinoma	Yes	[44]
Nuclear Imaging	PET	^11^C	Murine NK	Naïve	Mice	Intravenous	30, 60 min	Migrated to fibrosarcoma	Yes	[45]
	SPECT	^111^In-oxine	Primary NK	Naïve	human	Intravenous	1.5–144 h	Migrated to renal cell carcinoma	Yes	[28]
		^111^In-oxine	Primary NK	Naïve	human	Intraarterial	6, 24, 72, 96 h	Liver metastases with colon carcinoma	Yes	[27]
	AR	[^18^F] FDG	NK-92	Naïve	mice	Intravenous	30 min	Migrated to HER2/neu positive mouse sarcoma cell line	Yes	[48]
	GC	^99m^Tc-oxine	Primary NK	Naïve	mice	Intravenous	1–24 h	Migrated to thyroid cancer	Yes	[35]

FLI, fluorescence imaging; BLI, bioluminescence imaging; MRI, magnetic resonance imaging; PET, positron-emission tomography; SPECT, single photon-emission computerized tomography; NIR, near infrared; SPIO, superparamagnetic iron oxide; USPIO: Ultrasmall Superparamagnetic iron oxide; Fluc, firefly luciferase; AR, autoradiography; GC, gamma camera; NRP-body, NK-cell–recruiting protein-conjugated antibody.
